# Intermittent long-wavelength red light increases the period of daily locomotor activity in mice

**DOI:** 10.1186/1740-3391-3-8

**Published:** 2005-05-31

**Authors:** John R Hofstetter, Amelia R Hofstetter, Amanda M Hughes, Aimee R Mayeda

**Affiliations:** 1 Roudebush VA Medical Center, 1481 W. 10^th ^St., Indianapolis, IN, 46202, USA; 2 Berry College, P.O. Box 491640, Mt. Berry, GA 30149-1640, USA; 3 Richmond-upon-Thames College, Egerton Road, Twickenham, Middlesex, UK

## Abstract

**Background:**

We observed that a dim, red light-emitting diode (LED) triggered by activity increased the circadian periods of lab mice compared to constant darkness. It is known that the circadian period of rats increases when vigorous wheel-running triggers full-spectrum lighting; however, spectral sensitivity of photoreceptors in mice suggests little or no response to red light. Thus, we decided to test the following hypotheses: dim red light illumination triggered by activity (LEDfb) increases the circadian period of mice compared to constant dark (DD); covering the LED prevents the effect on period; and DBA2/J mice have a different response to LEDfb than C57BL6/J mice.

**Methods:**

The irradiance spectra of the LEDs were determined by spectrophotometer. Locomotor activity of C57BL/6J and DBA/2J mice was monitored by passive-infrared sensors and circadian period was calculated from the last 10 days under each light condition. For constant dark (DD), LEDs were switched off. For LED feedback (LEDfb), the red LED came on when the mouse was active and switched off seconds after activity stopped. For taped LED the red LED was switched on but covered with black tape. Single and multifactorial ANOVAs and post-hoc t-tests were done.

**Results:**

The circadian period of mice was longer under LEDfb than under DD. Blocking the light eliminated the effect. There was no difference in period change in response to LEDfb between C57BL/6 and DBA/2 mice.

**Conclusion:**

An increase in mouse circadian period due to dim far-red light (1 lux at 652 nm) exposure was unexpected. Since blocking the light stopped the response, sound from the sensor's electronics was not the impetus of the response. The results suggest that red light as background illumination should be avoided, and indicator diodes on passive infrared motion sensors should be switched off.

## Introduction

One of the earliest observations in the study of circadian rhythms was that continuous light (LL) lengthens circadian period in most nocturnal animal species [1]. "Aschoff's Rule" posits that there is a positive log-linear relationship between the LL intensity and period [2-5]. In all these studies LL was white light, in one study full-spectrum light [4]. However, we found that mice had slightly longer circadian periods when the monitoring device was a passive infrared (ir) proximity sensor compared to a system using ir beams that crossed the cage. The only obvious difference between the systems was that the proximity sensor had a small, red light-emitting diode (LED) that came on immediately after discerned motion and stayed on for several seconds after motion was not discernable.

The first question to be raised is whether a dim red LED can affect the circadian system of mice. The circadian rhythm of locomotor activity in rats is entrained by red light [6]. However, several studies which examined the spectral sensitivity of the photoreceptors in mice suggest little or no response to red light. The peak sensitivity of the photoreceptors that mediate phase shifts in pigmented inbred mouse strains is between 500 nm [7] and 511 nm [8] (blue-green light). The sensitivity of the photoreceptors drop sharply and is vanishingly small at wavelengths above 600 nm (orange light) [7-9]. In mice lacking rods and cones, the peak sensitivity for phase-shifting is 481 nm, and sensitivity drops to zero at less than 600 nm [9]. In pigmented mice, electroretinographic responses to a flickering monochromatic light and the behavioral responses to a forced-choice discrimination task peak at 510 nm [10]. The light sensitivity in both tests drops sharply as the wavelength approaches 600 nm. Melanopsin, in combination with the classical rod and cone photoreceptors, accounts for the transduction of photic information to the circadian system. We are unaware of studies of the Aschoff effect in rodless and coneless mice, but melanopsin knockout mice have an attenuated Aschoff effect compared to wild-type mice [11,12].

The second question is whether light presented only in response to activity can lengthen period in mice. Pittendrigh and Daan suggested that light pulses during the photosensitive portion of an animal's circadian cycle mimic the effect of LL on period [13]. Ferraro and McCormack (1986) confirmed this in rats using feedback lighting (LDfb) [14]. In their LDfb apparatus, the lighting in each rodent's cage was controlled by each rodent's own locomotor activity. When wheel revolutions reached a certain rate, the cage lighting came on. The lights went out 2 minutes after wheel-running tapered off below the target rate. They compared LL to LDfb at 0.1, 1 and 100 lux of light. They found that circadian period under LDfb obeyed Aschoff's rule, and feedback lighting increased circadian period by the same amount as an equivalent irradiance of continuous light.

In earlier studies we showed that C57BL/6 and DBA/2 mice differed in their Aschoff affect comparing constant dark to constant full-spectrum LL. At 10 lux, the C57BL/6 mice had an increase in period of 1.20 hours but the period of the DBA/2 mice increased only 0.20 hours [4]. Consequently we predicted that the C57BL/6 mice would have a greater increase in period under the LD feedback regimen than DBA/2 mice.

This study tests the hypotheses that a dim red LED provided as feedback to activity elicits an increase in circadian period of locomotor activity and that C57BL/6 and DBA/2 mice have a differential response to the red light stimulus.

## Methods

### General housing and care

Mice were housed singly in optically clear polycarbonate cages (L × H × W: 11 × 8 × 7 in) with approximately 250 ml of Sani-chip^® ^(Harlan Teklad) bedding. They were acclimated under alternating 200 lux light and dark of 12 hours each (LD 12:12) for at least two weeks prior to the start of the study. Food (Teklad 7001 Mouse & Rat Diet 4%) and water were continuously available throughout the study. All animals were maintained in facilities fully accredited by the Association for the Assessment and Accreditation of Laboratory Animal Care. All research protocols and animal care were approved by the Institutional Animal Care and Use Committee in accordance with the guidelines of the *Guide for the Care and Use of Laboratory Animals *(Institute of Laboratory Animal Resources, Commission on Life Sciences, National Research Council, 1996).

### Experimental housing and care

For measurement of circadian period, all test mice were kept in a sound attenuating, ventilated room at a constant temperature (23°C) and under continuous darkness (DD). Sound attenuating, opaque dividers were placed between the test cages. Caretakers wore a Pelican Versabrite headlamp fitted with a red safelight beam diffuser. The diffuser/filter transmitted light greater than 600 nm only. Care in the darkroom consisted of ten min per day and each mouse was inspected for less than a minute. Daily visits occurred at random times between 8 am and 5 pm.

### Locomotor activity assessment

Daily locomotor activity of the mice was monitored with passive infrared detectors (Ademco, Syosset, NY) mounted over each cage. The passive infrared (ir) proximity sensor works by emitting pulses of ir light, and then measuring the distance to objects from the flight time of the reflected signal. Whenever the distance changes, the detector opens or closes a switch. All detectors were tested to ensure response uniformity. Each detector had a red LED that switched on when motion was detected, then switched off 3 to 5 seconds after movement was no longer sensed. The LED could be disabled with a switch mounted on the motion sensor circuit board.

### Spectral analysis

The irradiance spectra of the LEDs were determined using an S.I. Photonics fiber optic spectrophotometer (Tucson, AZ). The distance between the fiber optic input probe and the LED was 18 cm (the depth of the mouse cage). A random sample of eight of the 16 motion sensors used in the study was assessed. A hand-held light meter (UDT Instruments, Baltimore, MD) was used to measure illuminance produced by the LED at about 5 cm from the bottom of the cage and 13 cm from the LED. The test was repeated with the LEDs covered with black electrician's tape.

### Assessment of circadian period of locomotor activity

Activity events were grouped into 5-minute bins by the Stanford Chronobiology Systems' (Stanford, CA) data processing and automatic storage system integrated into a Dell computer. Clocklab, the biological rhythm analysis software (Actimetrics, Evansville, IL), was used to calculate the period of locomotor activity of each mouse using linear regression through activity onsets of the last 8 to 10 days of each treatment.

### Experiment 1: Hypothesis – LEDfb changes circadian period compared to DD

#### Mouse husbandry

C57BL/6 mice were bred in our facility from mice purchased from Jackson Laboratory (Bar Harbor, ME). Two male and six female C57BL/6 mice between the ages of 185 to 295 d were studied.

#### Experimental protocol

Four mice were put under motion sensors with the LED enabled (LEDfb), and four were put under sensors with the LED disabled (DD). The locomotor activity of the mice was monitored for two weeks (Stage 1). Then treatment was switched, and activity of the mice was monitored for another two weeks (Stage 2). The circadian period for each stage was calculated from the last 8 to 10 days under a given condition.

#### Statistical analysis

A factorial ANOVA (SAS ver. 9.1) tested for effect of sex, age, lighting condition (LEDfb compared to DD) and sequence of light treatment (LEDfb first compared to DD first). A post-hoc Tukey's Studentized Range test compared periods under different lighting protocols.

### Experiment 2: Hypothesis – The effect of LEDfb on circadian period can be eliminated by blocking the light source

#### Mouse husbandry

Twelve male C57BL/6 mice aged 30 d were purchased from Jackson Laboratory and housed singly.

#### Experimental protocol

Following acclimation to our facility in LD 12:12 for two weeks, they were moved into DD. All mice were put under motion sensors with the LED enabled (LEDfb), but black electrician's tape covered the LEDs of six motion sensors (taped LED) for two weeks. For Stage 2, all the LEDs were turned off for two weeks of DD. For Stage 3, the treatment condition of Stage 1 was switched, i.e. all LEDs were enabled but the LEDs previously covered by tape were uncovered and the previously uncovered LEDs were covered. Mouse activity was monitored continuously throughout the experiment.

#### Statistical analysis

A one-way ANOVA tested for effect of lighting condition (DD compared to LEDfb and taped LED), with a post-hoc Tukey's Studentized Range test.

### Experiment 3: Hypothesis – C57BL/6 and DBA/2 mice have different response to LEDfb

#### Mouse husbandry

Eight male C57BL/6 mice and eight male DBA/2 mice were purchased from Jackson Laboratory, age 4 weeks.

#### Experimental protocol

Following acclimation to our facility in LD 12:12 for two weeks, mice were moved into DD. Four mice of each strain (DBA/2 and C57BL/6) were put under motion sensors with the LED enabled (LEDfb), and four of each strain were put under sensors with the light disabled (DD). At the end of Stage 1, they were moved to LD 12:12 for one week. When mice returned to the assessment room under DD, the treatment condition was switched. Activities of all mice were monitored for another two weeks.

#### Statistical analysis

A two-factor ANOVA tested for effect of strain and lighting condition (LEDfb compared to DD) with a post-hoc Tukey's Studentized Range test. The change in period from LEDfb compared to DD (Δτ) was calculated for each mouse and compared by a *t*-test.

## Results

### Light measurements

**Figure 1:**
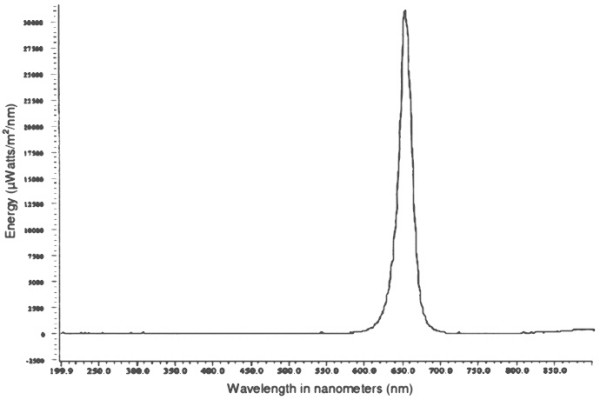
The irradiance spectrum of a red LED integrated into the passive-infrared motion sensor circuitry.

The irradiance spectrum of the LED for a sample of 8 proximity sensors was a narrow band centered on 652 nm. Figure 1 shows a representative spectrum. The illuminance of the LED was one lux. The illuminance of the LED covered with black electrician's tape was zero.

### Experiment 1: LEDfb changes circadian period compared to DD

**Figure 2:**
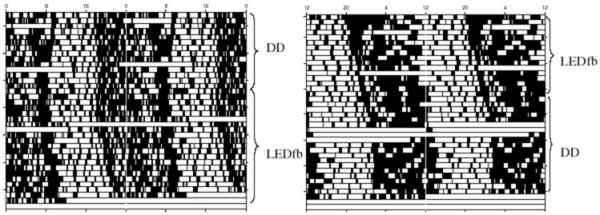
**Double-plotted actograms of C57BL/6 mice under DD and dim red LEDfb**. Lighting conditions are shown to the right of each actogram.

**Figure 3:**
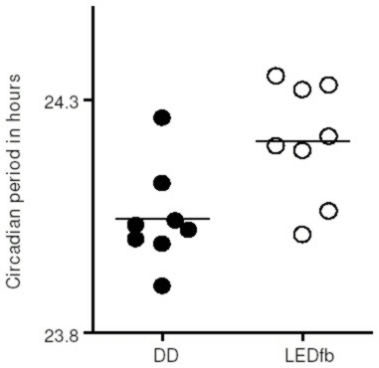
**The circadian period of C57BL/6 mice is longer under dim red LEDfb than DD conditions (p = 0.0095)**. Lines show the mean period for each group.

Representative actograms of C57BL/6 mice under DD and dim red LEDfb are shown in Figure 2. The mean period in DD was 24.05 ± 0.04 h, and under red light LEDfb it was 24.21 ± 0.04 h. A factorial ANOVA testing for effect of sex, age, lighting condition (LEDfb compared to DD) and sequence of light treatment showed no effect of sex, age or sequence but an effect of lighting condition on period [F_1,5 _= 7.72, p = 0.039]. A post hoc Tukey's test showed longer period with LEDfb compared to DD (p = 0.0095). Figure 3 shows the effects of LEDfb on the circadian period of locomotor activity.

### Experiment 2: The effect of LEDfb on circadian period can be eliminated by blocking the light source

**Figure 4:**
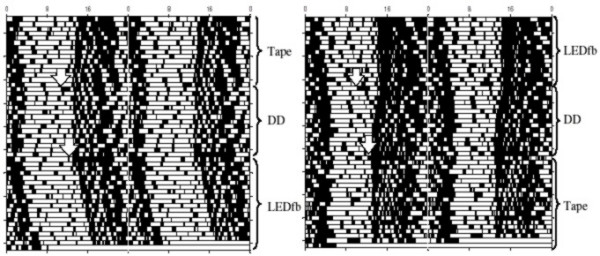
**Double-plotted actograms of C57BL/6 mice under DD, dim red LEDfb, and LEDs covered with black tape**. Lighting conditions are shown to the right of each actogram. Arrows show onset of new lighting conditions.

**Figure 5:**
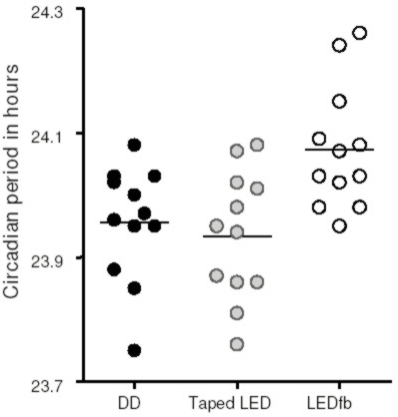
**Circadian period of C57BL/6 mice is longer under dim red LEDfb than when the light source is covered by black tape (p < 0.01) or DD (p < 0.025)**. Lines show the mean period for each group.

Representative actograms of C57BL/6 mice under DD, dim red LEDfb, and LEDs coved with black tape are shown in Figure 4. The mean period of 12 mice in DD was 23.96 ± 0.03 h; under taped LEDs, it was 23.93 ± 0.03 h; and, under LEDfb, it was 24.07 ± 0.03 h. There was a significant effect of lighting condition by one-way ANOVA [F_2,33 _= 7.02, p = 0.0029]. A post-hoc Tukey's test showed a longer period under the uncovered LED than under the tape-covered LED (p < 0.01) or DD (p < 0.025), as summarized in Figure 5. Periods did not differ between DD and tape-covered LED.

### Experiment 3: C57BL/6 and DBA/2 mice do not have different responses to LEDfb

**Figure 6:**
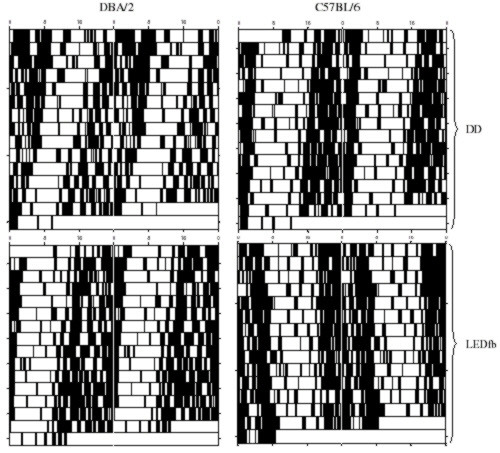
**Double plotted actograms of DBA/2 and C57BL/6 mice under DD (top) and dim red LEDfb (bottom)**. The actograms at top and bottom are from one DBA/2 mouse (left) and one C57BL/6 mouse (right). Lighting conditions were separated by two weeks of LD 12:12.

**Figure 7:**
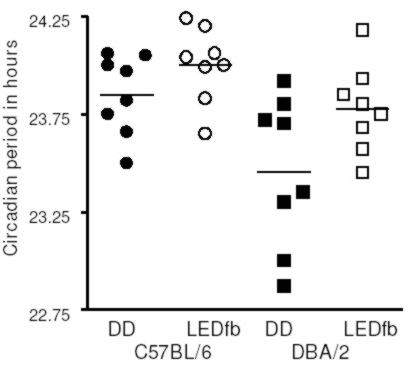
**The circadian period of both DBA/2 and C57BL/6 mice under DD (filled symbols) and dim red LEDfb (open symbols)**. Lines show the mean period for each group. Overall, mice had longer period under LEDfb than DD (p < 0.025), and C57BL/6 mice had longer periods than DBA/2 mice (p < 0.01).

Representative actograms of DBA/2 and C57BL/6 mice under DD and dim red LEDfb are shown in Figure 6. For C57BL/6 mice, the mean period under DD was 23.85 ± 0.07 h; the mean period under LEDfb was 24.00 ± 0.07 h. For DBA/2 mice, the mean period under DD was 23.46 ± 0.14 h; the mean period under LEDfb was 23.78 ± 0.08 h. A two-factor ANOVA testing for effect of strain and lighting condition (LEDfb compared to DD) showed a significant effect of both strain [F_1,14 _= 7.73, p = 0.0147] and lighting condition [F_1,14 _= 10.99, p = 0.0051] but no interaction. A post-hoc Tukey's test showed longer period with LEDfb compared to DD (p < 0.025). The C57BL/6 mice had different periods from the DBA/2 mice by post-hoc Tukey's test (p < 0.01). Figure 7 shows the effects of LEDfb on the circadian period of locomotor activity in the two strains of mice.

The mean increase in period with LEDfb (period under LEDfb minus period under DD, Δτ) for C57BL/6 mice was 0.15 ± 0.05 h. For DBA/2 mice, Δτ was 0.32 ± 0.13 h. The increase in period with LEDfb did not differ between strains by *t*-test (p = 0.26).

In summary, circadian period was significantly longer under LEDfb (a small, red LED whose intensity was about 1 lux which came on only when a mouse was active) than that under DD in both C57BL/6 and DBA/2 strains of mice. The LEDs gave off red light in a narrow band centered on 652 nm. Covering the LED with black tape blocked the effect of the dim red light. Furthermore, there was no difference in this effect between the two strains.

## Discussion

This study suggests that the circadian system in mice is responsive to long wavelength red light. The result is surprising because recent studies suggest that melanopsin, in combination with the classical rod and cone photoreceptors, account for the transduction of photic information to the circadian system. There are no studies of spectral sensitivity of the Aschoff effect in mice. However, the spectral sensitivity of photoreceptors mediating circadian phase-shifts in mice is vanishingly small at wavelengths above 600 nm [7-9]. In most organisms, circadian period under LL is a function of both intrinsic period and photic inputs. The Aschoff effect is understood to result from the cumulative phase-shifting effect of LL on the pacemaker [5,13,15]. Thus an effect of red light on circadian period is unexpected.

One possible explanation is that there is another photopigment present in mammals that is sensitive to far-red light and affects period rather than phase. It cannot be excluded that period and phase are affected by different light receptors or light receptive pathways. It seems more likely that low sensitivity to red light via the known circadian photopigments has a cumulative period-lengthening effect on the pacemaker. The timing of light exposure could have amplified this effect. Under LEDfb mice received light between circadian time (CT 12) and CT 24, during their active phase. The period-response curves (τRC) of mice have period-shortening between CT 4 and CT 16 and period-lengthening between CT 16 and CT 4 [16]. LEDfb should cause substantial period-lengthening, with minimal period-shortening.

This study has several limitations. Only the Aschoff effect was investigated, and this was not under the usual protocol of constant light. Nevertheless, the increase in circadian period of mice under LEDfb was consistent with a prior activity feedback study in rats, where wheel-running triggered full-spectrum illumination resulting in lengthened circadian period [14].

Covering the LED with black tape blocked the effect of the dim red light. We conclude that ultrasound from the LEDs or the electronics associated with their illumination did not produce the effect. Mice emit ultrasonic cries and this is important in maternal behavior [17-19]. However, it is unlikely that covering the lights with black tape would block ultrasound. Although this remains a possibility, we are aware of no instances in the literature where ultrasound causes either a phase response or a change in circadian period.

The C57BL/6 and DBA/2 mice did not differ in the amount period increased with 1 lux red light LEDfb. This is in contrast to an earlier study where C57BL/6 mice had a greater increase in period with 10 lux full-spectrum LL vs DD than DBA/2 mice [4]. One possible explanation is that DBA/2 mice are more sensitive to period-lengthening effects of red light than of full-spectrum light. Another possibility is that the shape of the τRC differs between the two strains, so that for C57BL/6 mice more of the period-lengthening portion of the τRC lies outside of the time when they received light during LEDfb than for the DBA/2 mice. In this case, constant LL would have more period-lengthening effect than LEDfb.

The results of this study suggest that investigators cannot use continuous dim red light to simulate DD, and must be judicious in using red "safe" lights for animal care in DD. Further studies are needed to determine whether constant red light of this output spectra and intensity lengthens period more than LEDfb, and whether it can phase-shift the circadian rhythms in mice.

## Conclusion

Mice under a dim, long-wavelength red light that came on intermittently when the animals were active had a circadian period that was long compared to their free-running period under DD. Covering the light with black tape blocked the response. Furthermore, under the conditions described, the magnitude of the mean circadian period increase in DBA/2 and C57BL/6 strains of mice was indistinguishable.

## Competing interests

The author(s) declare that they have no competing interests.

## Authors' contributions

JRH conceived of the study, participated in its design and statistical analysis and drafted the manuscript.

ARH carried out experiment 1, designed and carried out experiment 3, and wrote the Methods section.

AMH participated in the statistical analysis and designed and set up experiment 2.

ARM carried out experiment 2, participated in the statistical analysis and assisted in drafting the manuscript.

All authors read and approved the final manuscript.
